# Predicting molecular structure from sparse NMR data, proof-of-concept using a graph neural network

**DOI:** 10.1039/d6dd00189k

**Published:** 2026-09-17

**Authors:** Ben Honoré, Calvin Yiu, Jose Napolitano-Farina, Dave Russell, Iuni Margaret Laura Trist, Ruth Dooley, Craig P. Butts

**Affiliations:** a School of Chemistry, University of Bristol UK craig.butts@bristol.ac.uk; b Genentech Inc. USA; c Evotec(UK) Ltd Milton Park Abingdon UK

## Abstract

We demonstrate that graph-based neural networks are capable of molecular structure elucidation of organic molecules directly from experimentally measurable NMR spectroscopic properties, simulated by DFT. Such a system is a holy grail for researchers in chemical science for molecules in the solution state. Our graph-based transformer neural network architecture is based on ‘inverting’ IMPRESSION-G2 (which outputs predicted NMR properties based on input molecular structures) to instead output a predicted molecular structure in one shot from input computed NMR properties. The inclusion of scalar (*J*) coupling input NMR features is shown to be critical to this approach, correctly assigning ∼99.9% of bonds and correctly predicting >95% of molecules in one-shot when all theoretical *J* values are included (the ‘Everything’ model). When simulated NMR data are limited to only the experimentally realistic 1H and 13C NMR parameters (‘Experimentally measurable simulated parameters’ model), the success rate drops substantially to ∼10%. This can be improved by using multi-shot predictions combined with confidence metrics and systematic searching of heteroatom bonding structures to deliver 78.3% success rate on molecules with <2 nitrogens and <2 oxygens and 32.4% overall for the testing set used. This proof-of-concept demonstrates the potential to map spectroscopic properties directly onto molecular structure properties using graph neural networks and highlights key sensitivities to spectroscopic data sparsity and heteroatom degeneracy when taking this approach.

## Abstract 2

We demonstrate that graph-based neural networks are capable of molecular structure elucidation of organic molecules directly from experimentally measurable NMR spectroscopic properties, simulated by DFT.

## Graphical abstract

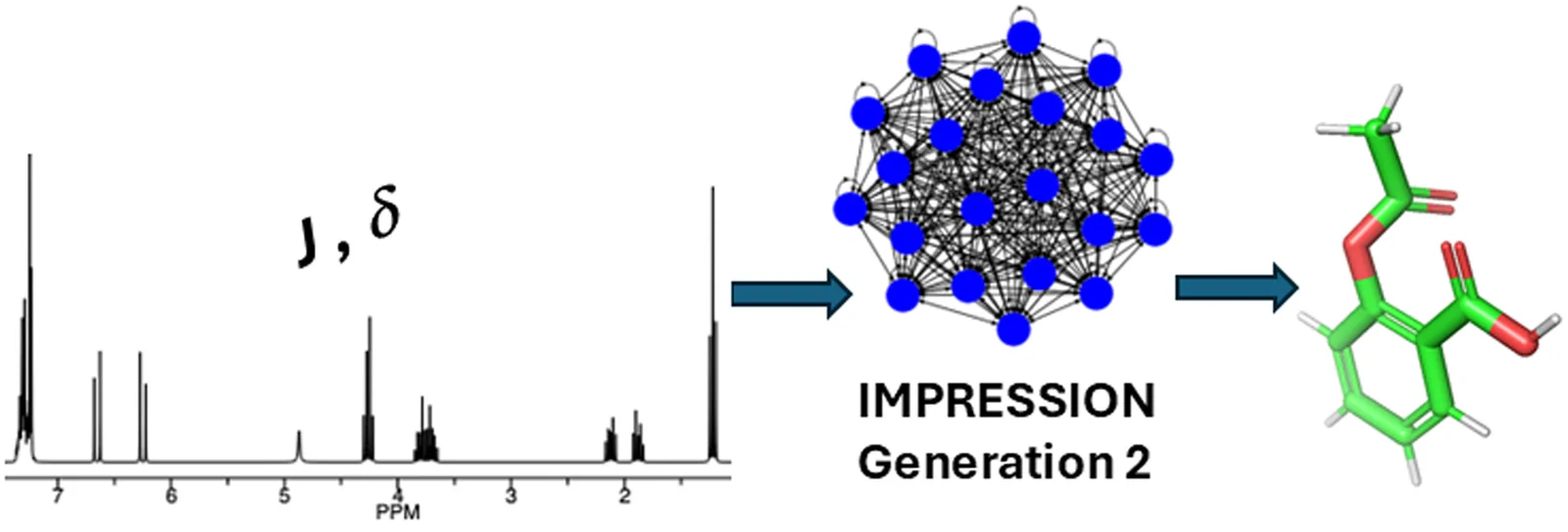

## Introduction

Nuclear magnetic resonance (NMR) spectroscopy is among the most powerful tools in chemical structure elucidation and a crucial part of chemical science, in academic research and the drug development pipeline for example. The atom-level detail provided by NMR means that in theory a chemist can piece together the 2D constitution, 3D configuration and 3D conformation of a molecule in solution from its NMR data. Interpreting NMR spectra, however, can be a challenging and slow task even for expert scientists, leading to incorrect answers where the sample is a large and complex structure or the spectroscopic data is limited or ambiguous. Generative machine learning provides a potential avenue to addressing this challenge.

Generative AI approaches are being shown to have benefits when attempting to predict chemical structures that have desirable properties *e.g.* good binding affinity to a protein target^1–3^ or superconductivity.^4,5^ However this is a relatively simple task - identifying any plausible chemical structure that exists within an area of “property space” *i.e.* selecting one of the millions of molecules that should share relevant properties with molecular structures on which the machine learning system is trained. However molecular structure elucidation is many orders of magnitude more complex – mapping sparse and exact spectroscopic information (*δ*^1^H and *δ*^13^C NMR chemical shifts for example) precisely to the one-and-only correct molecular structure that has those spectroscopic properties. In practice that means selecting the only single correct structure from the entirety of chemical space, estimated to be very loosely on the order of 10^60^ molecules.^6^

One can define the problem of predicting molecular structure from spectroscopic properties as the ‘inverse problem’ of predicting spectra from molecular structure. There are several approaches for this inverse problem being explored, including large language models^7–11^ and graph-based methods.^12–14^ LLMs represent a promising direction for mapping NMR data to molecular structure as they can efficiently manage large scale training sets (millions of molecules) but access to large, curated experimental datasets which contain more than chemical shift data at this scale is a challenge. We propose that information rich graph-based methods are capable of solving this task for large compounds (>10 heavy atoms) through a 1 : 1 direct mapping of NMR data to molecular structure. Using information rich graphs allows for much smaller datasets (the order of thousands of molecules + spectra), at which level, more information dense NMR training data can be feasibly generated.

Graphs are intuitive representations for both molecular structure and NMR spectra, consisting of nodes denoting atoms (or NMR chemical shifts) and edges denoting interatomic relationships such as bonding (or NMR coupling). Graph neural networks (GNNs) aggregate information from atom neighbours in a molecular graph and are scalable predictors of molecular properties,^15^ such as spectra, and can efficiently train on datasets of many thousands of molecules. Furthermore, by incorporating interatomic distances as edge features, graph representations can capture 3D structure and stereochemistry with rotational invariance. In the context of NMR spectroscopy, this has enabled the development of several successful graph-based machine learning predictors of (atomic/node) NMR chemical shift properties and (interatomic/edge) *J* coupling constants in NMR prediction.^16–19^ However, graph-based approaches have largely struggled to solve the inverse task *i.e.* predicting molecular structure from NMR data. Existing graph-based attempts to directly solve exact molecular structure with machine learning are limited to reduced scales of the challenge *e.g.* by working with very small molecules, for example the QM9 dataset of ∼130k molecules – a theoretical dataset of HCNOF compounds containing no more than nine heavy atoms.^20^ At this scale, the problem becomes amenable to brute-force approaches, *e.g.* Monte Carlo tree search.^12–14^ However such stepwise approaches do not scale well to larger and more diverse molecules, with exponential explosion of the chemical space size being explored for each extra heavy atom added to the structure. For example, the GDB-17 dataset^21^ of molecules comprising 17 or fewer C, N, O, S and halogens contains over 166 × 10^9^ structures, one million times more than QM9. Another means of reducing the size of a molecular search space is to work with molecular fragments as opposed to individual atoms, thus reducing the number of bonds that need to be predicted.^10,22^ However, given a real-world search space closer to 10^60^ molecules, it is clear that brute-force methods cannot be a generalisable solution.

Previous graph-based approaches have tended to focus exclusively on *δ*^1^H and *δ*^13^C chemical shifts; at this stage we suggest that the sparsity of information available through chemical shifts and their similarity within isomeric clusters makes the problem hugely underspecified, and potentially impossible to solve uniquely *i.e.* the exact molecular structure cannot be computed solely from NMR chemical shift data in every case. However, chemical shift is not the only type of information available from NMR spectral data and herein we leverage scalar (*J*) coupling information to exploit the 1 : 1 mapping of molecular structure graph (atoms and bonds) to spectrum graph (chemical shifts and coupling constants) and thus predict, in a supervised learning approach, correct bonding structures of compounds of sizes which become of interest to for example pharmaceutical applications.

We base our approach on the ‘inversion’ of our recently reported IMPRESSION-G2 (Intelligent Machine PREdiction of Shift and Scalar Information Of Nuclei, Generation 2)^19^ – a graph transformer neural network model that outputs NMR data based on an input molecular structure. IMPRESSION-G2 achieves the forward prediction of multiple classes of NMR parameters simultaneously (chemical shifts and *J* coupling constants) for all nuclei in an input chemical structure. To modify IMPRESSION-G2 for our purposes, we can simply switch the input and output channels of the model, so it outputs structure from input spectroscopic data. Another way to view this is to consider the forward task as a formulation of computing *f*_forward_, as per:NMR data = *f*_forward_ (Bonding structure, *M*_f_)where *M*_f_ is the molecular formula (measurable by mass spectrometry). With formula and bonding information already separated in the IMPRESSION-G2 graphs, we can thus define the inverse problem – predicting structure from spectrum – as determining *f*_inverse_:Bonding structure = *f*_inverse_ (NMR data, *M*_f_)

We demonstrate here this proof-of-principle, that inversion of IMPRESSION-G2 generates a machine learning system that can predict structure from the NMR parameters extracted from NMR spectra. We demonstrate that the inverse problem is underspecified as a chemical shift-only graph-based approach but that including NMR *J* coupling information in the input graphs makes the problem soluble in a much expanded chemical space.

## Method

The overall workflow of converting NMR parameters into molecular structures is shown in Fig. 1, which is the exact inverse of the workflow used in our original IMPRESSION-G2 architecture.^19^ First NMR parameters are converted into a graph using the same structure as IMPRESSION-G2 (atomic number and chemical shifts as node properties, scalar coupling constants as edge properties), which is passed through the graph transformer network to convert these into an output graph comprising atomic numbers as node properties and interatomic distances as edge properties. In effect, the inverted IMPRESSION-G2 graph transformer network is acting as an edge (distance) predictor. Finally, the chemical bonding, in the form of a connectivity matrix, is generated by defining a bond between all pairs of atoms (nodes) connected with an appropriately short edge distance (<1.8 Å in this case). All other atom pairs were designated as having no bond between them.

**Fig. 1:**
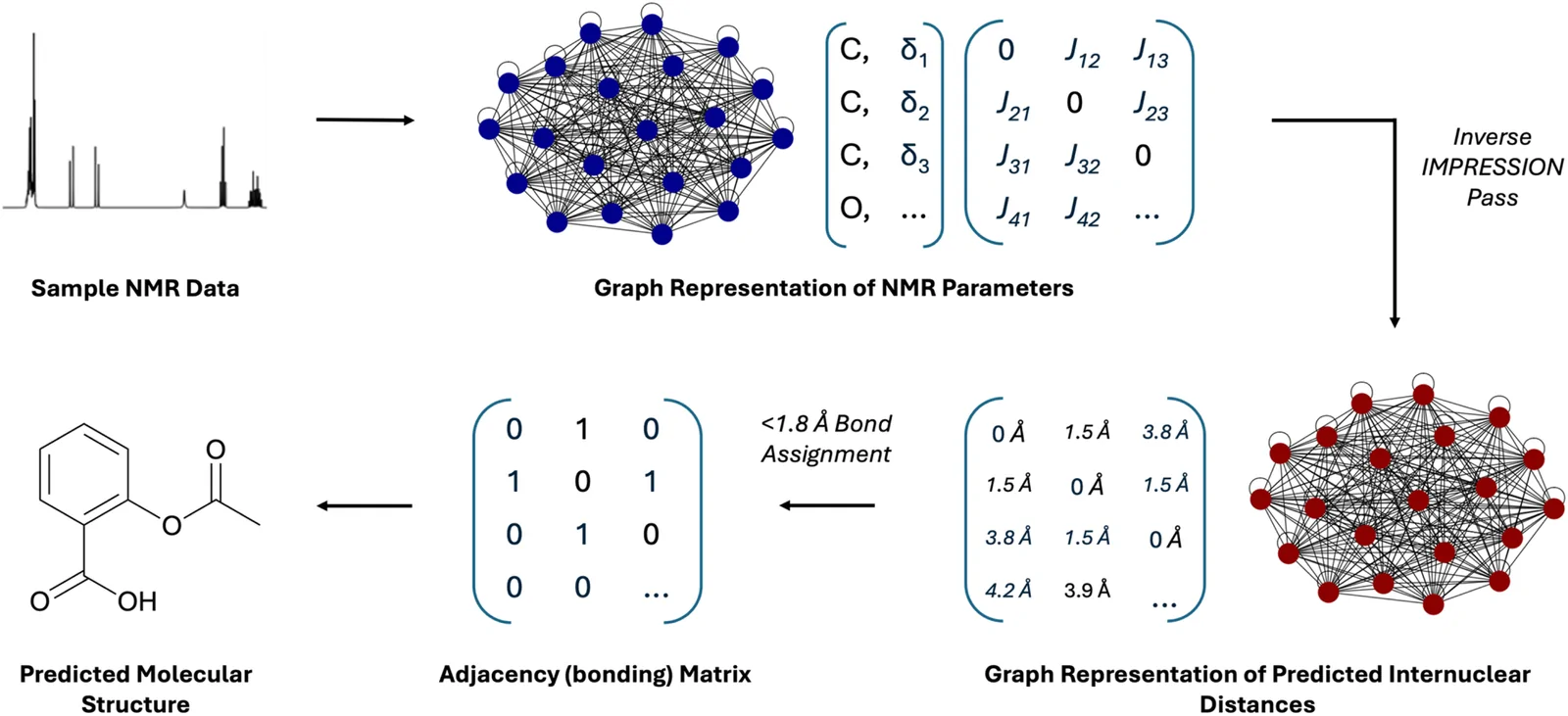
Prediction workflow of the inverted-IMPRESSION architecture.

In order to train the inverse-IMPRESSION graph transformer network, we used the subset of 15 502 fully connected molecular graphs which contained H, C, N, O and F atoms from the training dataset of the forward IMPRESSION-G2 model.^19^ These pharmaceutically relevant structures were originally sourced from the CSD,^23^ ChEMBL,^24^ the OTAVA Chemicals diversity library^25^ and NMRShiftDB,^26^ with more detail on selection criteria available in the original publication. The internuclear distances and NMR parameters for these molecular graphs were simulated by DFT-calculations from a single 3D structure for each molecule, generating a complete set of all NMR isotropic chemical shift and scalar coupling parameters for all nuclei and internuclear pairs. The DFT methods used were mPW1PW91/6-311g(d,p) for geometry optimisation, and ωB97X-D/6-311g(d,p) for GIAO NMR calculation.^27–30^ It should be noted that synthetic (DFT) data were chosen for this study in order to provide a robust proof-of-concept for conversion between NMR parameters and structures, enabling all possible parameters to be tested. Using synthetic data avoids accuracy in the NMR data and corresponding assignments, as well as enabling us to train a machine on a diverse range of chemical space, without being limited by access to physical samples or literature data.

The full database of graphs was split 90 : 10 into a training set of 13 964 molecules (548 021 *δ* parameters and 25 287 556 *J* parameters, 25 287 556 interatomic distances) and a test set of 1538 molecules (61 766 *δ* parameters and 2 907 672 *J* parameters with 2 907 672 interatomic distances).

Chemical shift and atomic number were included as input node features, and coupling constants were included as the input edge feature between pairs of atoms. No additional information was encoded onto the graphs that either directly or indirectly described the bonding between the two *e.g.* path length, bond order, *etc.* During training, interatomic distance was calculated as an edge target feature, with a log normal loss function centred at ∼1.2 Å to maximise accuracy for short (bonded) interatomic distances at the cost of accuracy for longer (non-bonded) distances. The models were trained for 1200 epochs to optimise for this (training curves and other details are available in SI).

## Results and discussion

The first test conducted was to train a model on all DFT-computed NMR parameters (the ‘Everything’ model) using the full training set of 13 964 molecules. The resulting predicted distances for the holdout test set of molecules agree with the true interatomic distance (Fig. 2). This agreement demonstrates that the relationship between NMR data and molecular structure can be successfully mapped through interatomic distance. The weighted loss function used during training successfully optimised the accuracy for short distances (<2 Å) providing a Mean Absolute Error (MAE) of 0.03 Å. Conversion of the predicted distances into molecular structures was readily achieved by simply assigning all distances that were <1.8 Å as ‘bonds’. This ‘Everything’ model performs very well, correctly predicting the entire molecular structure for >95% of molecules in a single 1-shot prediction test (Table 1, ‘Everything’). The test molecules have on average ∼42 bonding interactions between ∼40 atoms (21 non-H) and an average mass of 290.3 Da, so this task is very challenging and reflects ∼99.9% of all bonds being correctly identified by the model. However, the ‘Everything’ model is unrealistic in an experimental setting as it includes many NMR parameters that are experimentally very challenging or impossible to measure in solution-state NMR *e.g.* scalar coupling parameters between pairs of low isotopic abundance nuclei (^13^C–^13^C, ^13^C–^15^N and ^15^N–^15^N); NMR parameters for the quadrupolar ^17^O nucleus; and small (<2 Hz) scalar coupling constants.

**Fig. 2:**
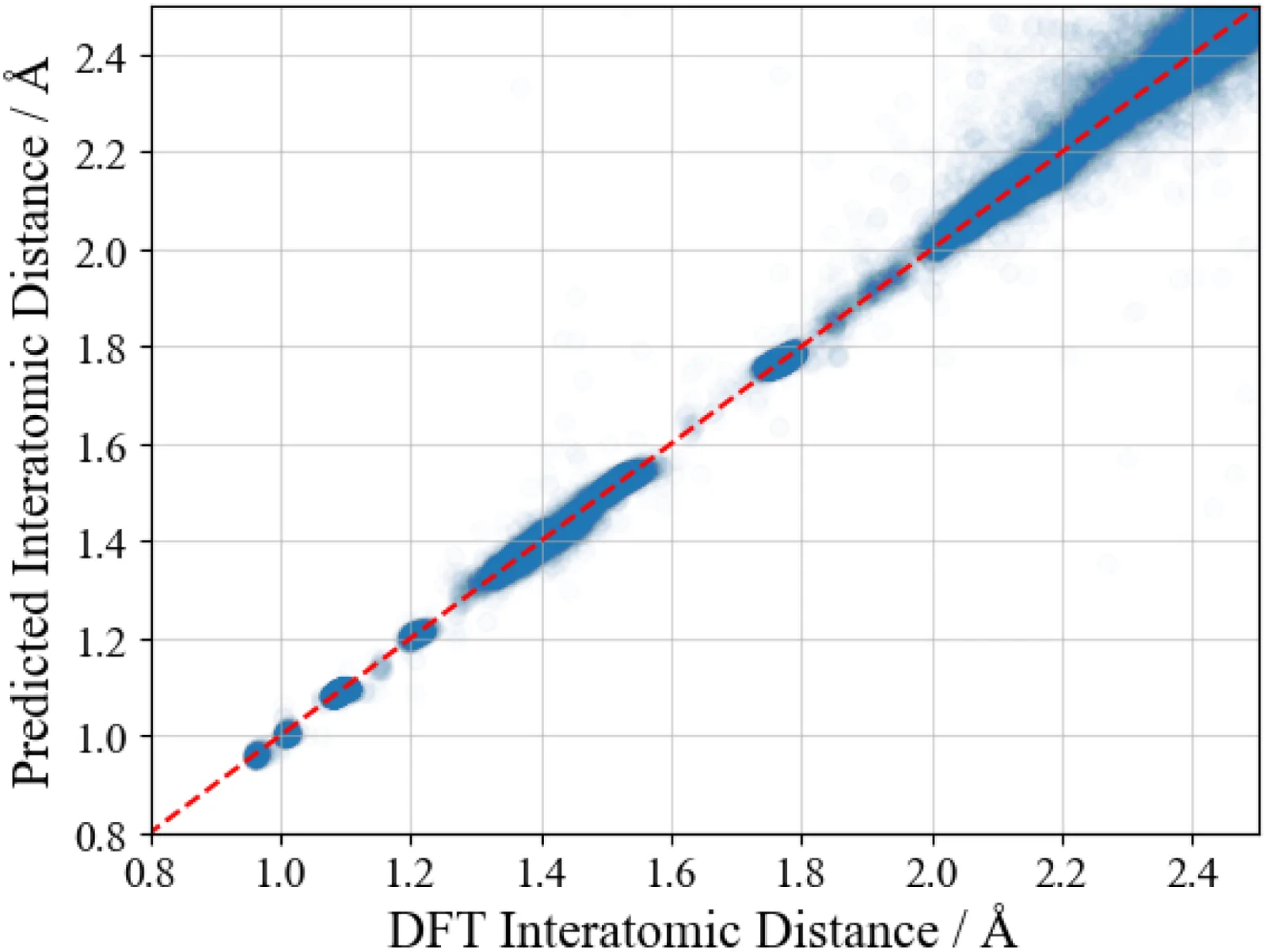
Accuracy of short-range (<2.5 Å) distances predictions from the ‘Everything’ model.

**Table 1:** Inverse-IMPRESSION test results on 10% hold-out set vary with input NMR features

Model	NMR atomic input features	NMR interatomic input features	1-Shot success rate	Found in 10 predictions	Ranked top in 10 predictions
‘Everything’	*δ* ^1^H, *δ*^13^C, *δ*^6^N, *δ*^17^O, *δ*^19^F	*J*_CHNOF–CHNOF_	95.6%	—	—
‘Experimentally measurable simulated parameters’	*δ* ^1^H, *δ*^13^C	*J*_CH–H_ >2 Hz	10.4%	12.7%	12.5%
‘Chemical shift only’	*δ* ^1^H, *δ*^13^C	N/A	0%	0%	0%

Therefore, a second more experimentally relevant model was trained on the same compounds but using parameters that would be commonly measured in routine solution-state NMR experiments. The parameters selected were: *δ*^1^H and *δ*^13^C chemical shifts and ^1^H–^13^C, ^1^H–^1^H scalar coupling constants that have values of >2 Hz (483 221 *δ* and 1 648 238 *J* with 25 287 556 target interatomic distances). As expected, the 1-shot molecular success rate for this experimentally relevant model drops substantially, to 10.4% (Table 1, ‘Experimentally measurable simulated parameters’) although again in this challenging task, ∼90% of all bonds are still correctly identified across the test set. Interestingly a third ‘Chemical Shift Only’ model, was trained and tested but failed entirely to correctly assign a single structure in the hold out test, highlighting that *J* coupling information is critical for inverse structure elucidation in this graph-based framework.

More detailed examination of the experimentally relevant prediction results showed that quite a number of structures (11.9%) were only slightly wrong – with <4 bonds differing from the correct structure. The model can be induced to predict alternative structures by making further predictions using slightly noised input NMR features *i.e.* by modifying the input chemical shifts and coupling by a small amount (0–1% for chemical shifts, 0–5% for couplings in our testing). With multiple candidate structures in hand, one can then deploy standard structure verification tools to establish a confidence score for each of those generated candidates. Here we used our forward IMPRESSION-G2 NMR-predictive model to allow comparison of the candidate structures' forward-predicted chemical shifts against the input values of the inverse model. See SI for full details on how the confidence metric was calculated (eqn (S6)).

When 10-shot prediction of every test molecule was used, the forward IMPRESSION-G2 confidence scoring categorised the correct structure as the top-1 most likely structure out of those 10, 98% of the time (when the correct structure was present in the set of 10 candidates). Ultimately, applying this ‘10-Shot’ approach increased the success rate of the ‘Experimentally measurable simulated parameters’ model only modestly, capturing the correct structure within 10 predictions 12.7% of the time, with the confidence metric successfully identifying the correct structure 12.5% of the time (Table 1).

Further consideration of the test cases where the model failed, reveals that model performance is highly sensitive to the number of heteroatoms and, more specifically, the number of heteroatoms of the same element. The success rate obtained on compounds containing 0 or 1 heteroatom is substantially higher (1-shot: 65.5%; 10-Shot: 78.3%) than when the molecule has 2–3 heteroatoms (both <2%), as shown in Table 2. This arises from the feature degeneracy of heteroatoms in the ‘Experimentally measurable simulated parameters’ model *i.e*. the heteroatoms have no input chemical shifts or coupling constants, and thus all heteroatoms of the same element type (N, O, F) in the molecule have identical properties as far as the network is concerned. Degenerate input atoms/nodes are thus predicted to have degenerate output distances/edges *i.e.* all degenerate heteroatoms are assigned identical bonding patterns as each other. This is exemplified by the two oxygens of a methyl ester, where the ‘Experimentally measurable simulated parameters’ model predicts both oxygens to bond to the methyl group (proposed structure, Fig. 3a). The prediction task using this approach is therefore essentially impossible for any molecule containing more than one of the same heteroatom (which defines the majority of the test set used here) unless those heteroatoms are entirely equivalent in their bonding structures, for example, the three fluorine atoms in a CF_3_ group – accounting for all the ∼1.5% structures correctly predicted with this model.

**Table 2:** ‘Experimentally measurable simulated parameters’ model performance depends on the number of heteroatoms in the test structure

Test subset	No. of molecules in test set	1-Shot success	Ranked top in 10-shot predictions	Ranked top by 10-shot and enumeration
Max. 1 of each heteroatom	235	65.5%	78.3%	78.3%
2–3 degenerate heteroatoms of 1 element	454	1.3%	1.8%	57.3%
2–3 degenerate heteroatoms of >1 element	218	0.0%	0.0%	24.8%
>3 degenerate heteroatoms of any element	631	0.0%	0.0%	0.0%
Overall	1538	10.4%	12.5%	32.4%

**Fig. 3:**
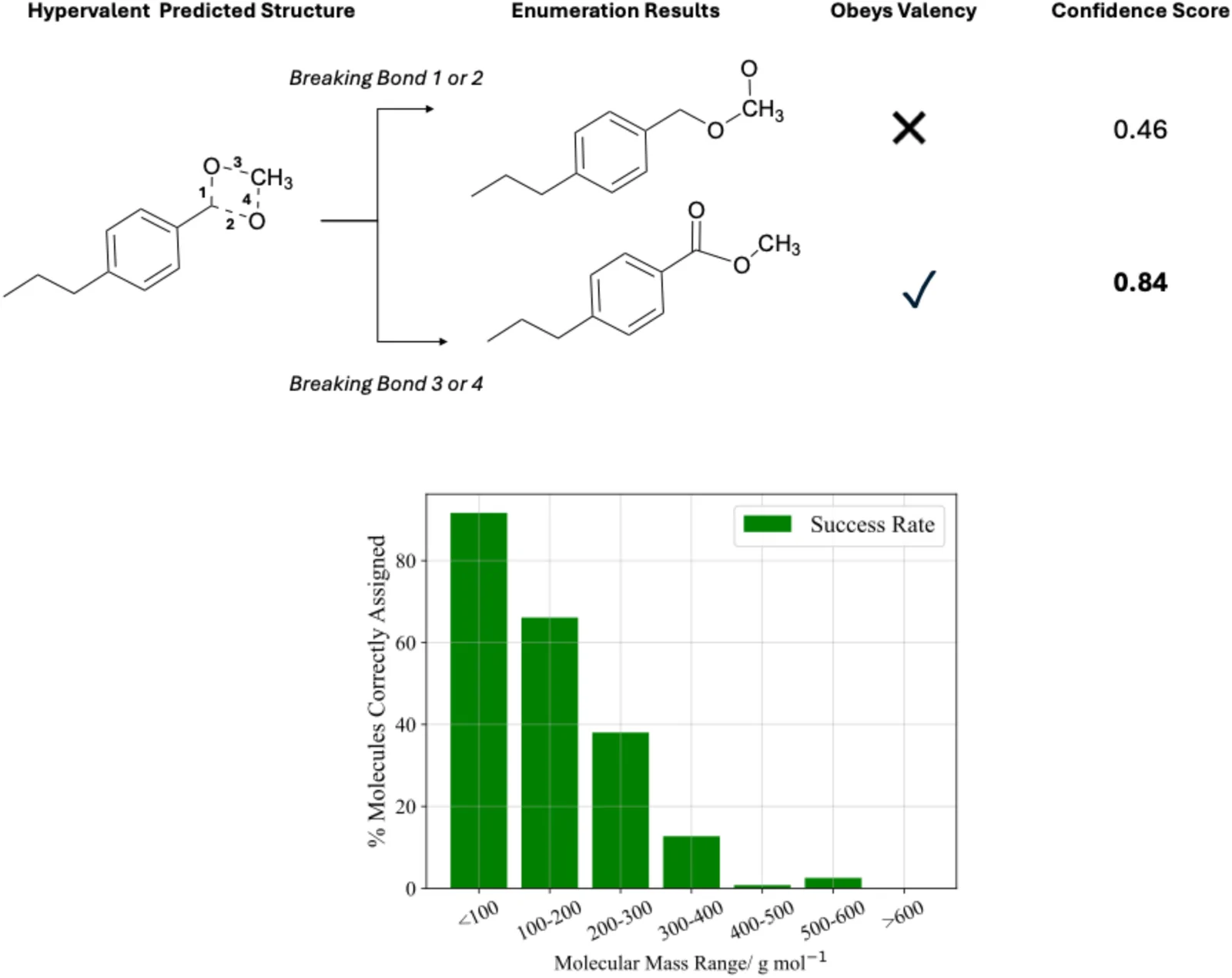
(a) The equivalent treatment of degenerate heteroatoms can be addressed by enumeration, and (b) how success rate varies with *M*_r_ (bottom).

We noted that this degenerate behaviour leads to predicted structures that do still contain the correct bonds (as well as false bonds) to the heteroatoms. It is thus plausible to methodically extract all chemically sensible structures from the hypervalent predictions by sequentially deleting bonds to enumerate all valent structures from the original predictions. Fig. 3a illustrates this for the case of a methyl ester. Here there are only two possible structures (deleting either bonds 3 or 4, or deleting bonds 1 or 2), and the second candidate structure has a substantially lower confidence score after chemical shift prediction (as well as being ruled out based on valency in this case). Combining this enumeration approach – with candidates ranked by the forward model – and 10-shot prediction gave a 57.3% success rate in retrieving the correct structures for test molecules with 2–3 instances of any given heteroatom. However, this brute force enumeration approach scales poorly when there are more than 3 degenerate heteroatoms in the structure, made worse by chemical valency *i.e.* nitrogen (trivalent) has a larger search space than oxygen (divalent).

This scaling makes enumeration exponentially more time-consuming for molecules with more heteroatoms and structure verification impractical in most of those cases. So by combining 10-shot prediction with enumeration, and confidence assessment using IMPRESSION-G2, the true structures are ranked as top-1 by the ‘Experimentally measurable simulated parameters’ model for 32.4% of the 1538-molecule hold out test set.

The performance of this model can be seen to be correlated to molecular mass (Fig. 3b). This is rationalised by the increasing number of bonds that need to be predicted, combined with the increased likelihood of degenerate heteroatoms in the molecule. One obvious improvement here would be to include NMR data for more heteroatoms *e.g.*^15^N data are perfectly plausible to measure, although not routinely used as they give approximately 3× weaker NMR spectra than ^13^C. Examples of molecules for which the model is successful (Fig. S9) suggest that the model is able to elucidate molecules of a complexity comparable to those which an experienced chemist would be expected to elucidate given sufficient NMR data, which is encouraging as to the value of graph neural network approaches to structure elucidation in this way.

## Conclusions

We have demonstrated the proof-of-concept that solving molecular structures from NMR parameters, using supervised machine learning with graph-based neural networks is both possible and efficient. The model performs with near perfect success when all possible *J* coupling and chemical shift data are included in the input feature parameters, demonstrating that the 1 : 1 mapping of molecular structure to simulated NMR data can be learnt. Limiting the input features to only experimentally accessible ^1^H and ^13^C simulated data reduces model performance, but combining this with multi-shot predictions, a structural enumeration stage for hypervalent degenerate heteroatom solutions and confidence scoring, delivers 32.4% success for the test set used here.

Looking ahead, testing on experimental data is a priority and will require treatment of conformational flexibility alongside dealing with variabilities arising from sample environment *e.g.* solvent. In this context we note the need here for robust experimental datasets which capture both chemical shift and scalar coupling data robustly. Integrating additional modalities of input spectroscopic data, including other NMR data (2D methods, NOEs, T1/2), infrared and mass spectrometry will also be expected to improve performance. With such robust data, we believe graph-based neural networks will provide a plausible and effective solution to the challenge of structure determination from NMR spectroscopic data.

## Author contributions

B. H. designed the inverse model's code, trained and tested the models, designed the enumeration and validation/confidence methods, co-designed the project with C. B. C. Y. built the core forward model code used and advised on the inverse model throughout. J. N.-F., D. R., I. T., R. D, supported development and co-supervised C. Y. and B. H. C. B. designed, developed and led the IMPRESSION research programme and supervised C. Y. and B. H. The manuscript was co-written by C. B. and B. H. with edits and comments from other authors.

## Conflicts of interest

There are no conflicts to declare.

## Supplementary Material

DD-005-D6DD00189K-s001

## Data Availability

Data, datasets, models and executable code are available at https://doi.org/10.5281/zenodo.20775496 and in the supplementary information (SI). Supplementary information: details on methods, architecture, training and testing data. See DOI: https://doi.org/10.1039/d6dd00189k.
