# Supplementary material for: Predicting molecular structure from sparse NMR data, proof-of-concept using a graph neural network
Source: Digit Discov. 2026 Sep 17. Online ahead of print. doi: 10.1039/d6dd00189k (PMC13647041; doi:10.1039/d6dd00189k)
Supplement: Supplementary file 1 [file DD-OLF-D6DD00189K-s001.pdf]

Supplementary Information (SI) for Digital Discovery.
This journal is © The Royal Society of Chemistry 2026

Predicting molecular structure from sparse NMR data, proof-of-concept using a graph neural network

Ben Honoré,*[a] Calvin Yiu,[a] Jose Napolitano-Farino,[b] Dave Russell,[b] Iuni Margaret Laura Trist,[c] Ruth Dooley,[c] and Craig Butts[a]

Supplementary Information

Table of Contents

Methods 2
- Graph Transformer Network 2
- Graph Representation 2
- Lamb Optimiser 2
- Log Normal Distribution Loss Function 2
- Hyperparameters 4
- Determining Bonding Structure 4

Training and Testing Data 5
- Databases 5
- DFT Methods 5

Results and Discussion 6
- Inverse-IMPRESSION Training Curves 6
  - Inverse-IMPRESSION Trained on all Theoretical NMR Parameters 6
  - Inverse-IMPRESSION Trained on Experimentally Accessible NMR Parameters 7
  - Inverse-IMPRESSION Trained on δ^1H and $\delta^{13}C$ 7
- Inverse-IMPRESSION Predicted Interatomic Distance Results 8
  - Inverse-IMPRESSION Trained on all Theoretical NMR Parameters 8
  - Inverse-IMPRESSION Trained on Experimentally Accessible NMR Parameters 9
  - Inverse-IMPRESSION Trained on δ^1H and $\delta^{13}C$ 10

Confidence Estimation 10

Performance Relative to Training Set Size 13

References 14

Methods

Test datasets, executable code and models for Inverse IMPRESSION are available here: https://doi.org/10.5281/zenodo.20775496

Graph Transformer Network

Inverse IMPRESSION is built with a Graph Transformer Network (GTN) architecture. In this approach, attention is applied over the nodes of the molecular graph to allow weight the information aggregated at each node according to its neighbours when predicting interatomic distances. The weighted sum of value vectors provides a contextual representation for the node pair that captures relational information in the graph. Because the distance between nodes is the target property, this model uses a single attention head, using only one relational weighting per pair of nodes. Multiple attention heads are unnecessary here since there is no additional complementary relational signal for the attention mechanism to exploit. This implementation of the GTN is an adapted version of the solution presented in the CHAMMPS Kaggle competition and deployed in IMPRESSION-G2[1].[2]

Graph Representation

A graph representation (*G*) is used to encode molecular formula and NMR information, and to predicted interatomic distances. Atoms are represented by of node (*V*) feature vectors and atom pairs are represented as a set of edges (*E*), which corresponds to:

$$G=(V, E) \quad \textbf{Eq. S1}$$

The feature information encoded onto each node is atomic number and chemical shift/shielding tensor. If chemical shifts of that class of atom are not included in the model training, the chemical shift feature is included as zero. An edge is encoded between every pair of atoms in the molecule. J coupling information is included as edge feature information; similarly, where certain J values are excluded, on the basis of being experimentally unmeasurable, the edge J value in the graph is encoded as zero. Interatomic distance is predicted on every atom pair as an edge target feature.

Lamb Optimiser

We used the Lamb optimiser for the training optimisation for inverse-IMPRESSION. This allows the optimiser to assign different learning rates per layer, providing improved stability and adaptive updates per parameter to prevent drastic changes in step sizes. In general, it has been shown to improve scalability of both batch sizes and model layer depth.

Log Normal Distribution Loss Function

We use a log normal function is compute the contribution of each interatomic distance prediction to the loss function. This is done to prioritise the model's consideration of atom

pairs at short distances as it is these predictions that defines the predicted bonding structure. μ is set to 0.4 and σ is set to 0.4 to generate the log normal distribution shown below that is approximately centred 1.25 Å. These are hyperparameters that were optimised during model development.

$$\omega(y_{E,\,true}) = \frac{1}{y_{E,\,true}\sigma\sqrt{2\pi}}\exp\left(-\frac{\left(\ln\left(y_{E,\,true}\right)-\mu\right)^2}{2\sigma^2}\right) \quad , \quad y_{E,\,true} > 0 \qquad \textbf{Eq. S2}$$

$$L = w(y_{E,\,true}) \times MSE(y_{E,\,pred}, y_{E,\,true}) \qquad \textbf{Eq. S3}$$

$y_{E,\,true}$ = true interatomic distance at edge *E*

$y_{E,\,pred}$ = inverse-IMPRESSION predicted interatomic distance at edge *E*

$\omega(y_{E,\,true})$ = weight given to atom pair $y_{E,\,true}$

σ = 0.4

μ = 0.4

L = calculated loss

MSE = mean squared error function

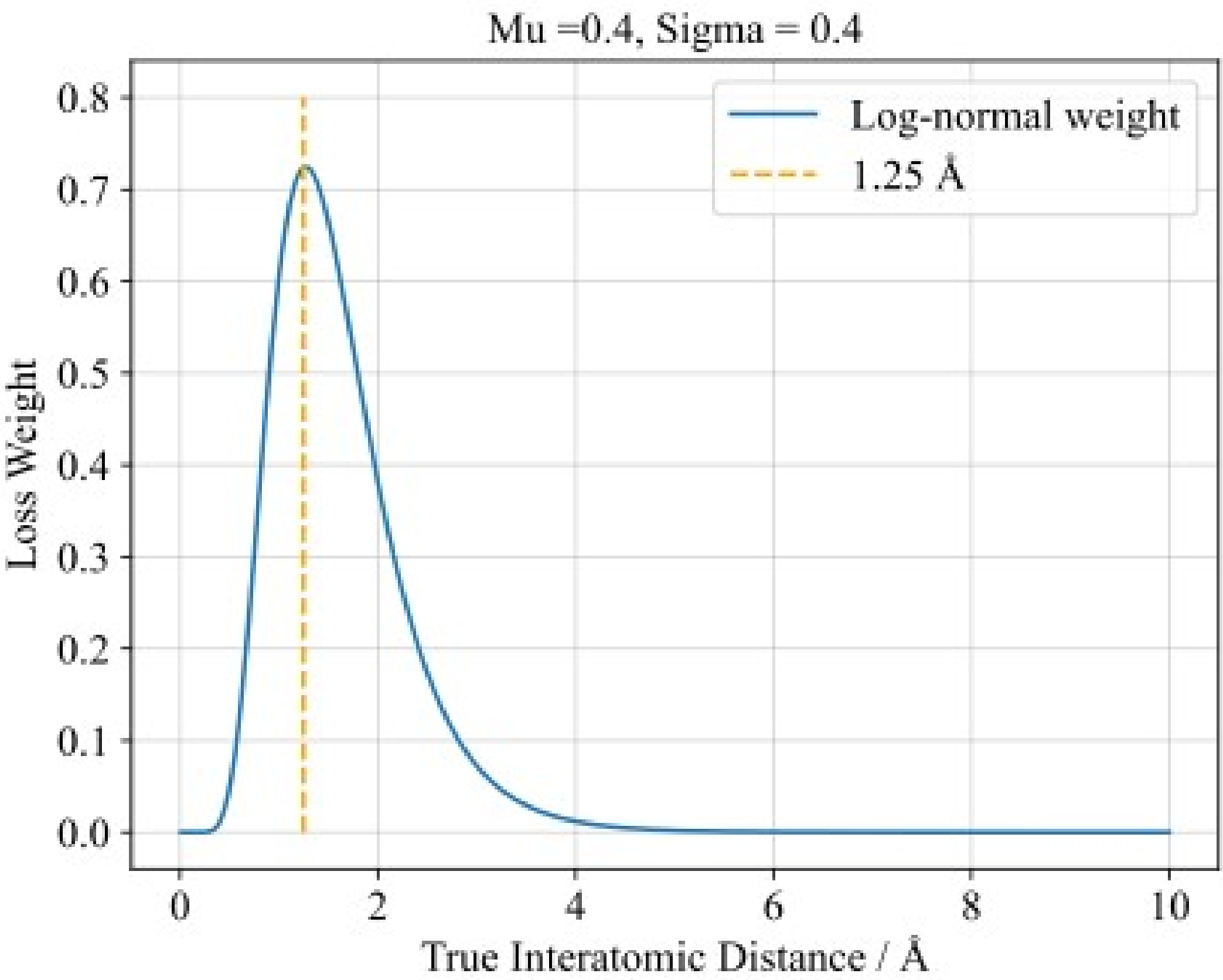

Fig S1. Log normal distribution centred on 1.25.

Hyperparameters

- Training epochs: **1200**
- Number of attention heads: **1**
- Number of layers (attention block): **4**
- Effective batch size: **400** (batch size, **50** * gradient accumulation, **8**)
- Target(s): **Interatomic Distance**

Training epochs are how many full passes of training data the model sees; 1 attention heads correspond to the single-headed attention mechanism; layers are the repeating block in the transformer architecture; effective batch size is the number of graphs (molecules) the model sees at a time before updating weights and also how many graphs the models sees at one time during prediction – this is calculated through the batch size multiplied by the gradient accumulation value; targets are the edge labels to be predicted.

Determining Bonding Structure

Bonds between atoms are defined by applying the basic assumption that any pair of atoms predicted to be closer than 1.8Å from one another are predicted as being bonded such that:

$$y_{E,\,predicted\ bond} = 1\ if\ y_{E,\,pred} < 1.8,\ 0\ if\ y_{E,\,pred} \geq 1.8 \quad \textbf{Eq. S4}$$

The threshold 1.8Å was selected based on the distribution of bonding and non-bonding interatomic distances on the model training data.

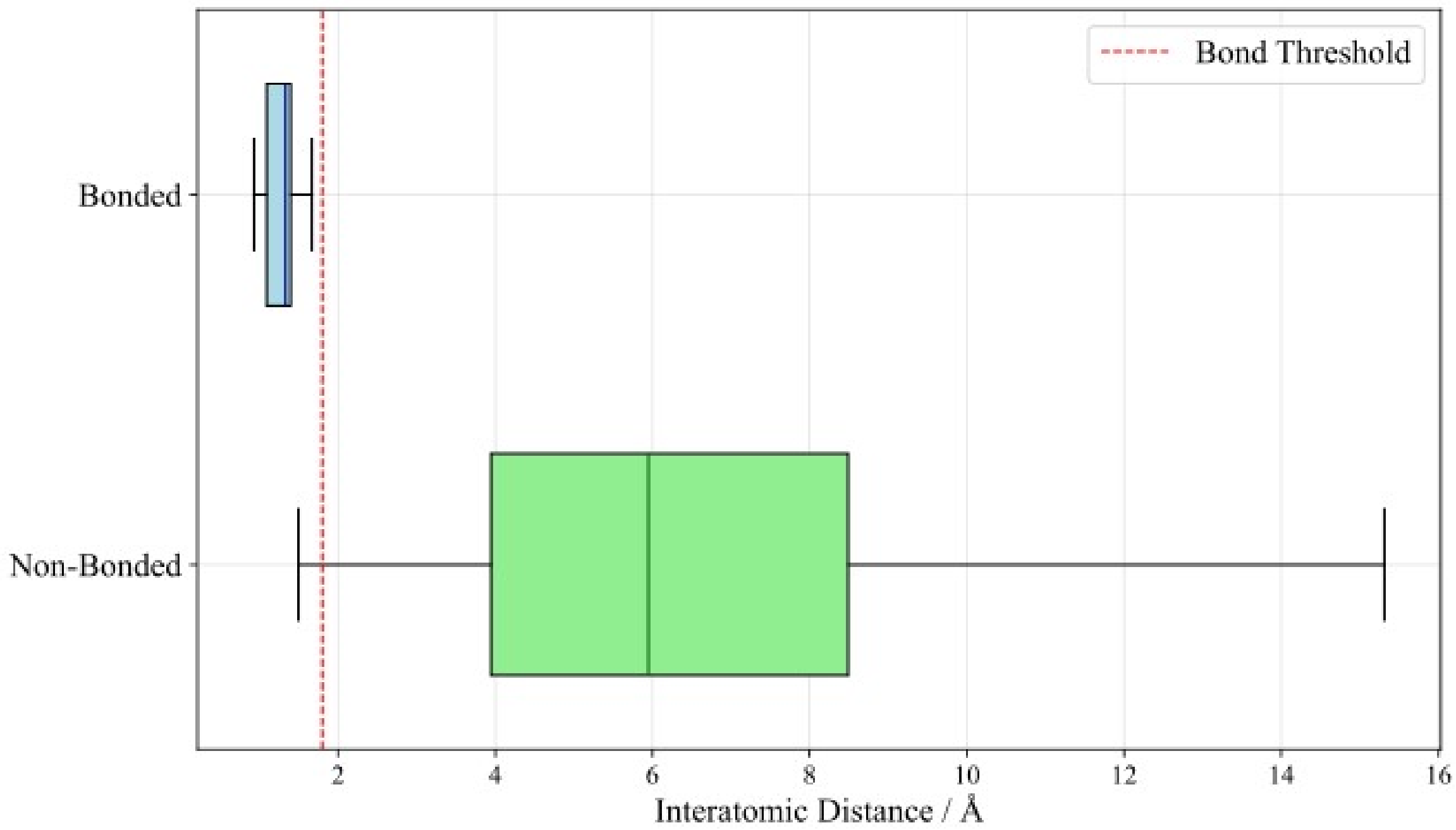

Fig S2. Distribution of bonded and non-bonded interatomic distances across test set.

Training and Testing Data

Databases

All molecules we use in our training data were sampled from large databases. These consist of Cambridge Structural Database (CSD)[3], ChEMBL[4], OTAVA[5] and NMRShiftDB[6].

Dataset Development and Preparation

The dataset was developed in instalments – the full workflow for database sampling is outlined in the IMPRESSION-G2 publication[1]. Active sampling (described in detail in ref. 6) was deployed to sample the first IMPRESSION dataset from the CSD. For further dataset expansion, a Tanimoto similarity comparison was carried to compare newly sampled structures (from the CSD and ChEMBL) against the existing IMPRESSION dataset, selecting for novel and underrepresented structures to maximise diversity. The full OTAVA dataset was incorporated into the IMPRESSION dataset as it had been designed with the express objective to maximise chemical diversity.[7]

The criteria for adding structures sampled from the above databases into the IMPRESSION dataset are as follows.

- Molecules must not contain any atom type outside of H, C, N, O, F, Si, P, S, Cl, Br.
- Molecules must not be charged.
- Molecules must be single fragments.
- Molecules must not already exist in the IMPRESSION dataset.

These criteria were evaluated in RDKit[8], with a Tanimoto fingerprint similarity comparison used to determine molecule degeneracy.

The IMPESSION datasets diversity-sampled from CSD, ChEMBL, OTAVA were supplemented by structures from the DFT8K dataset reported in the 2021 CASCADE publication[9]. The structures were re-optimised and NMR-computed by our DFT workflow and are available at https://github.com/Buttsgroup/IMPG2-testing-data/. The breakdown of the full inverse IMPRESSION dataset constitution is shown in Table S1.

Source	Train	Test	Total
CSD	7,301	373	7,674
NMRShiftDB	4,550	520	5,070
ChEMBL	2,113	257	2,370
OTAVA	0	388	388
**Total**	**13,964**	**1,538**	**15,502**

Table S1. Breakdown of data sources involved in dataset.

A 15,502 subset of molecules containing only H, C, N, O and F nuclei were taken from the existing dataset to train inverse IMPRESSION. This subset was randomly split 90:10 to obtain the training set (13,964 molecules, 548,021 δ parameters and 25,287,556 *J* parameters, 25,287,556 interatomic distances) and test set (1538 molecules, 61,766 δ parameters and 2,907,672 J parameters with 2,907,672 interatomic distances). This means that the test set represents the same distributional chemical space as the training set; however, the preprocessing and data preparation involved in developing the IMPRESSION dataset ensures no duplicated molecules and therefore a random train:test split removes the risk of data leakage.

DFT Methods

DFT Geometry Optimisation

Following initial geometry optimisation (Molecular Mechanics Section S1.3.2.1 for the generation of our training molecules) as highlighted in the main article, we carried out DFT geometry optimisation using Gaussian16 to get refined input coordinates. Here are the starting block and route line parameters used for all DFT geometry calculations for the training/testing data we generated:

```
%Chk=(molecule_name)_OPT.chk
%NoSave
%mem=26GB
%NProcShared=8
# opt=tight freq mPW1PW/6-311g(d,p) integral=ultrafine MaxDisk=50GB
```

DFT Tensor NMR Calculation

The optimised geometries are then extracted from the Gaussian16 output log files and used as input coordinates for the DFT NMR Tensor and Coupling Constant calculations. Here are the following block and route line parameters used for all DFT NMR parameters calculation for the training/testing data we generated:

```
%Chk=(molecule_name)_NMR.chk
%NoSave
%mem=26GB
%NProcShared=8
#T nmr(giao,spinspin,mixed) wB97XD/6-311g(d,p) maxdisk=50GB
```

Chemical Shift Scaling of DFT Tensors

As DFT calculates tensors, the values need to be linearly scaled to their chemical shift counterpart. This is done through comparison of DFT tensors against experimentally measured chemical shifts and calculating the slope (m) and linear correction (c) typical of the y = mx+c equation of a linear line, where x is the tensor and y is the chemical shift.

$$\delta EXP = \sigma DFT - cm\delta \mathrm{EXP} = \sigma \mathrm{DFT} - \mathrm{cm} \qquad \textbf{Eq. S5}$$

For ^{1}H and ^{13}C, we used the CHESHIRE reference set to calculate our scaling factors which were.

- ^{1}H = -1.0594, 32.2293 for m, c
- ^{13}C = -1.0207, 187.4436 for m, c

^{15}N and ^{19}F were gathered from literature with matching functional group and basis set[10, 11].

- ^{15}N = -1.0139, -148.67 for m, c
- ^{19}F = -1.0940, 173.02 for m, c

Results and Discussion

Inverse-IMPRESSION Training Curves

Inverse-IMPRESSION Trained on all Theoretical NMR Parameters

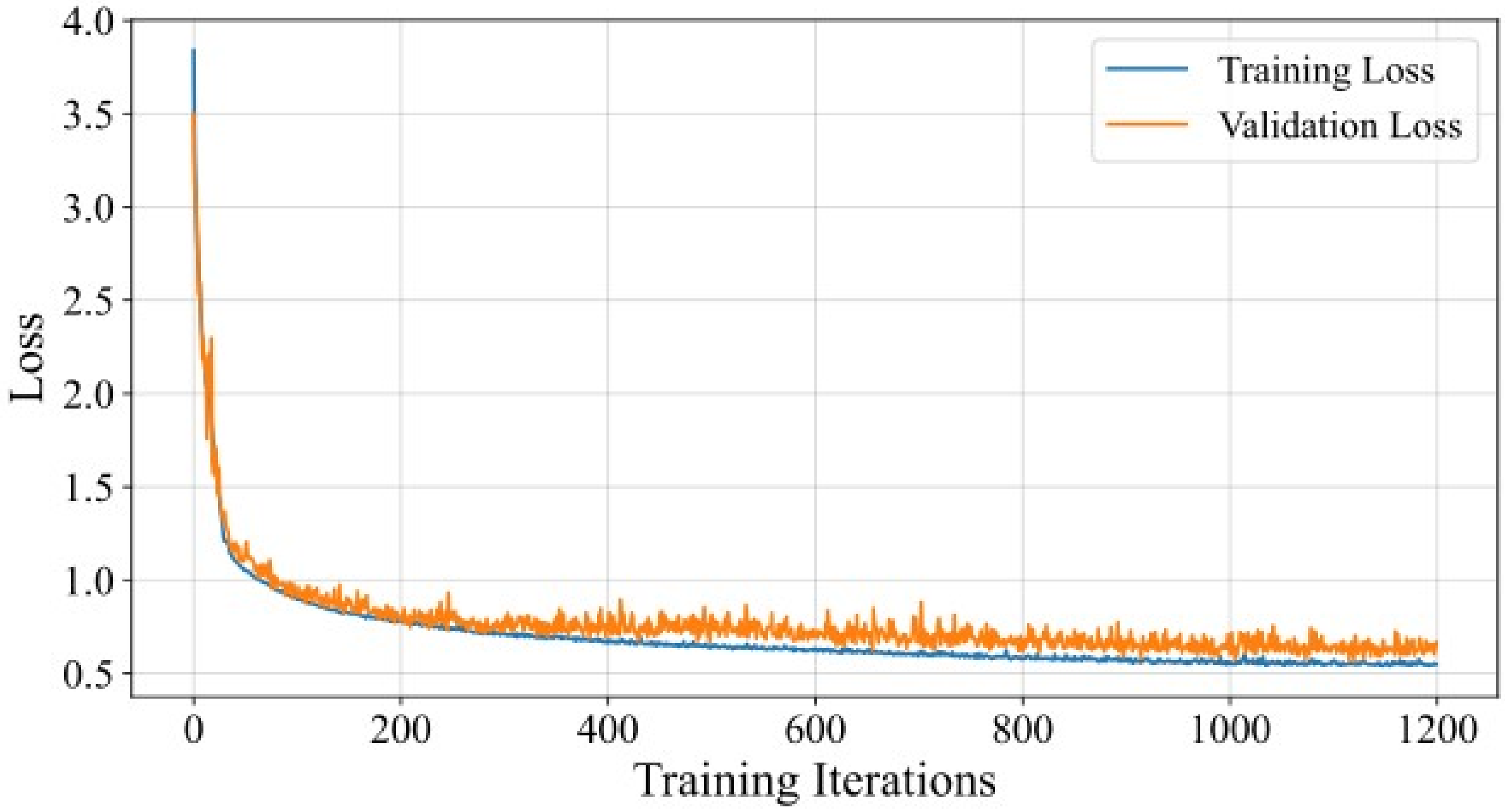

Fig S3. Training and test set loss changing with training epochs (all NMR parameters included).

Inverse-IMPRESSION Trained on Experimentally Accessible NMR Parameters

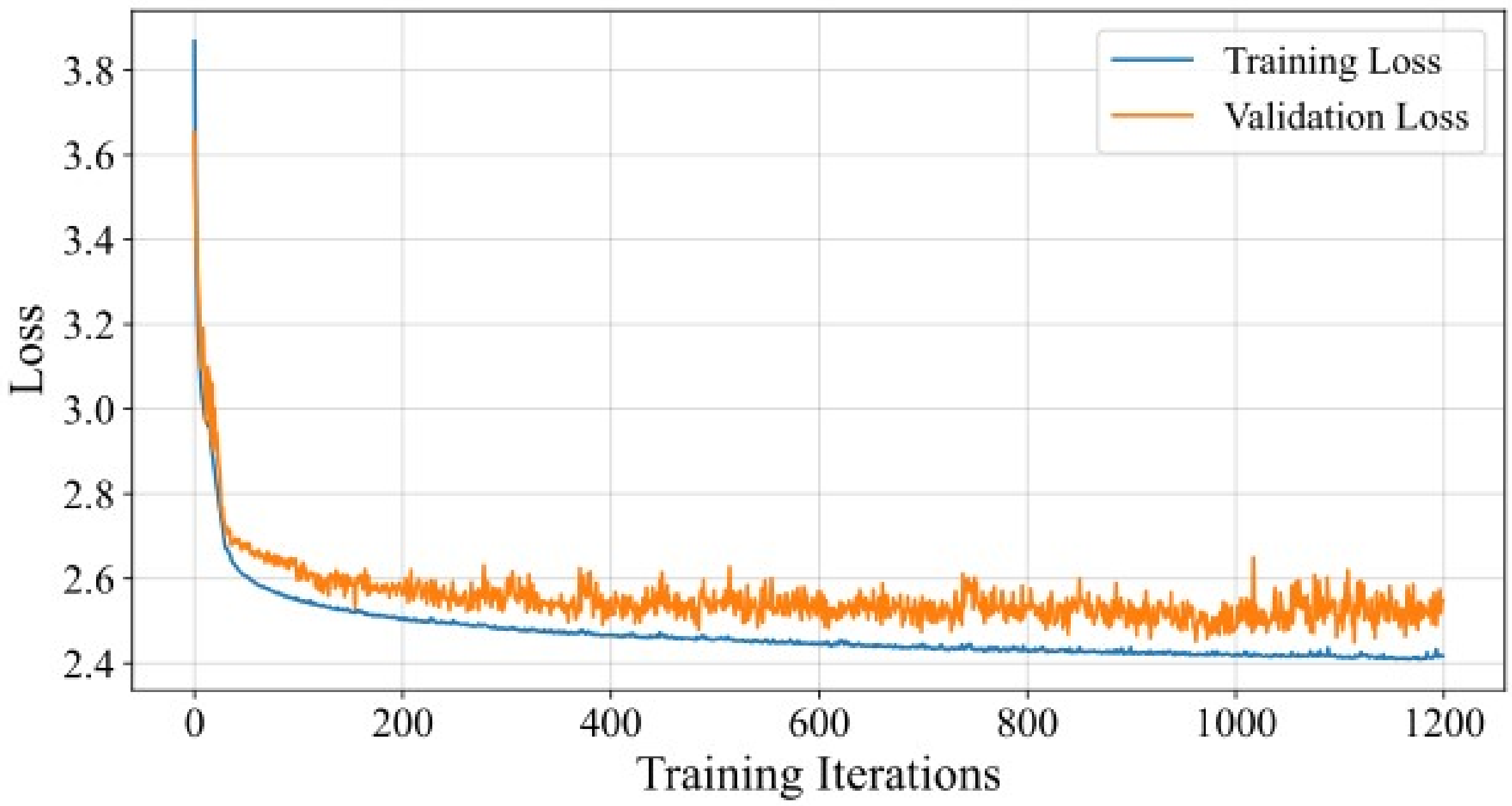

Fig S4. Training and test set loss changing with training epochs (experimentally accessible NMR parameters included).

Inverse-IMPRESSION Trained on δ^{1}H and δ^{13}C

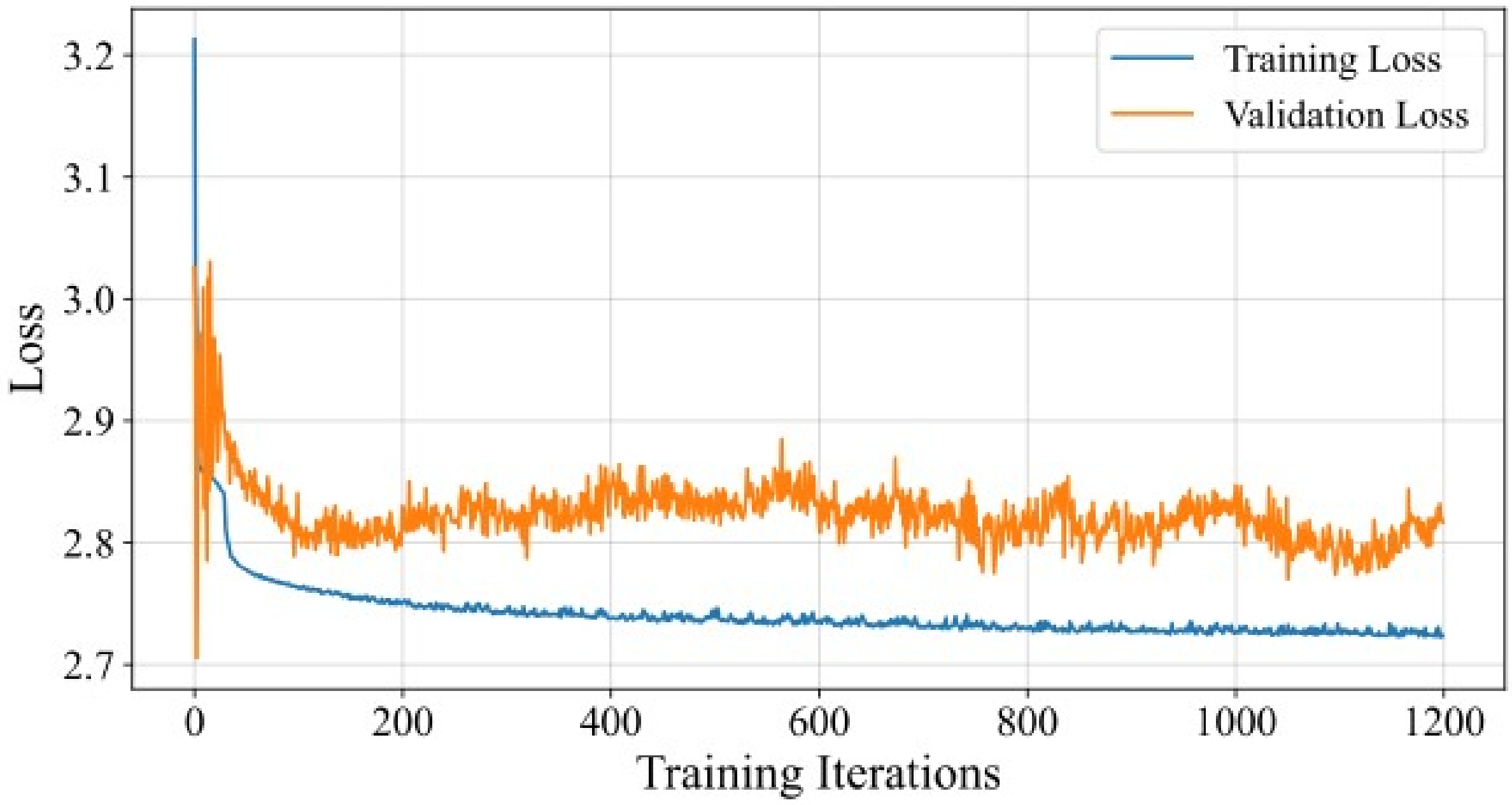

Fig S5. Training and test set loss changing with training epochs (just δ^{1}H and δ^{13}C chemical shift included).

Inverse-IMPRESSION Predicted Interatomic Distance Results

Inverse-IMPRESSION Trained on all Theoretical NMR Parameters

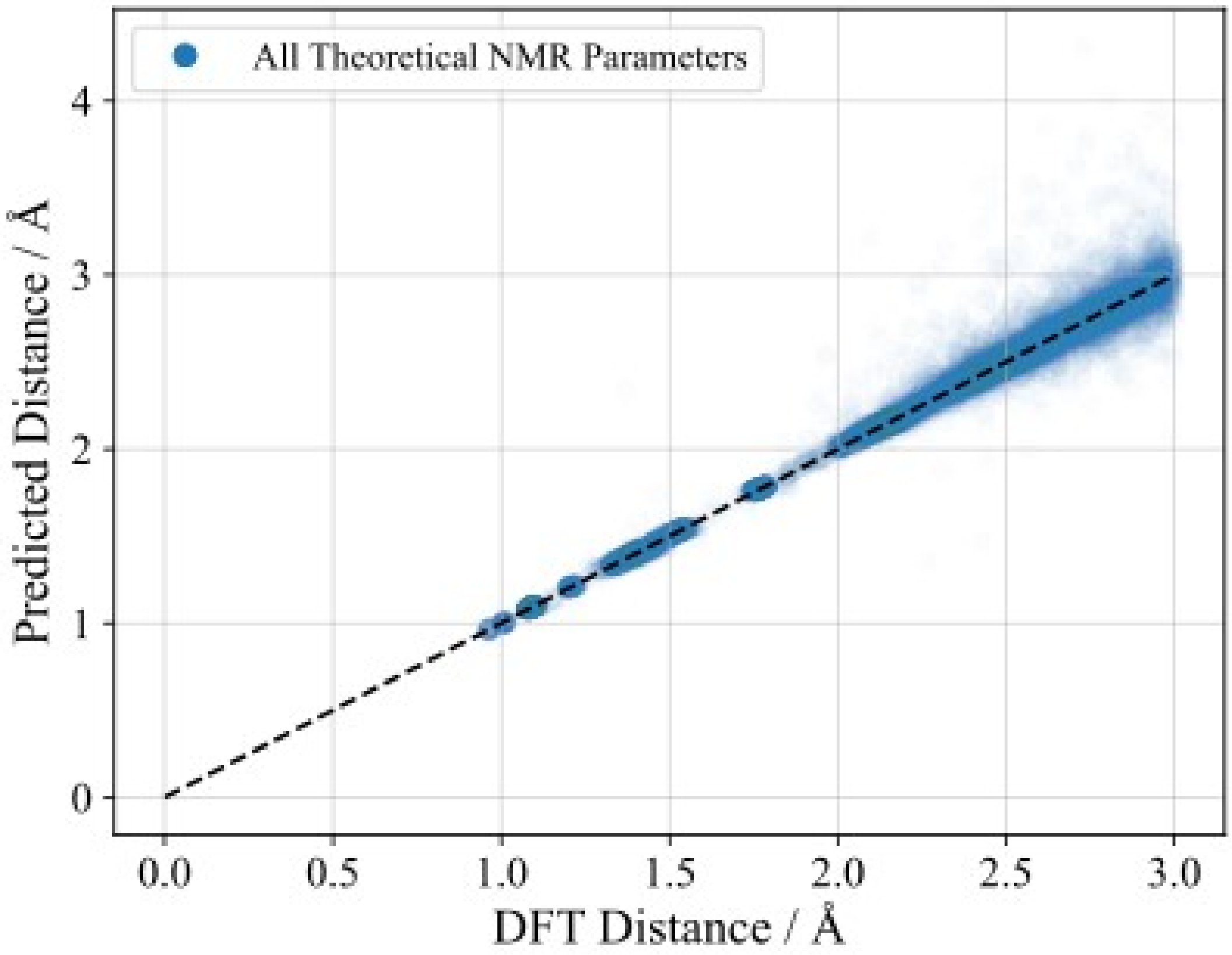

Fig S6. Predicted distances at short range (<3Å) when all NMR parameters included.

	MAE / Å	RMSE / Å	Mean Error	Max Error	R^2
All Distances	0.64	1.59	-0.53	22.3	0.79
Distances <Å	0.03	0.07	0.007	2.53	0.98

Table S2. Predicted distance error summary when all NMR parameters included.

Inverse-IMPRESSION Trained on Experimentally Accessible NMR Parameters

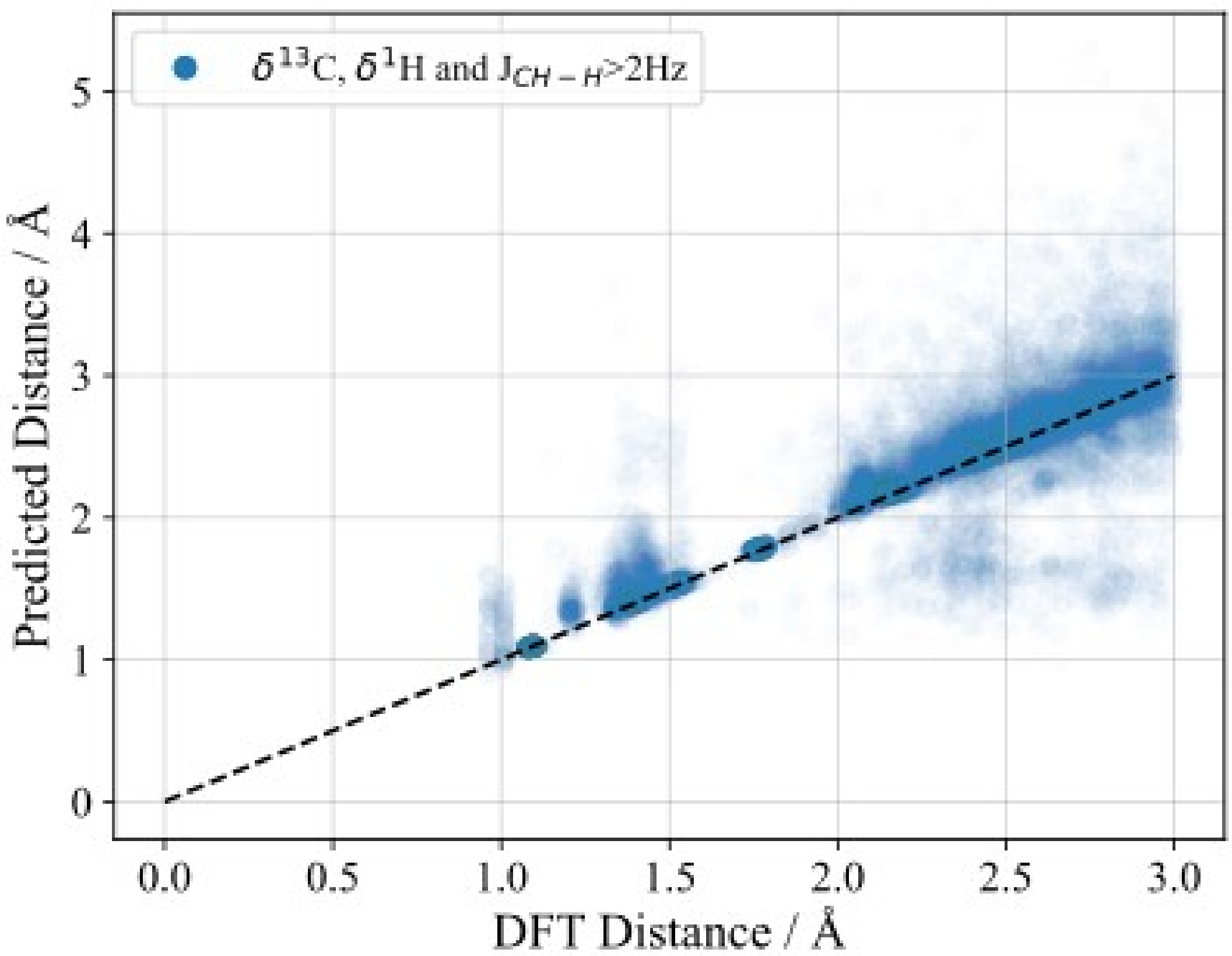

Fig S7. Predicted distances at short range (<3Å) when experimentally accessible NMR parameters included.

	MAE / Å	RMSE / Å	Mean Error	Max Error	R^2
All Distances	2.52	3.92	-2.45	26.9	-0.26
Distances<3Hz	0.12	0.25	0.04	2.90	0.80

Table S3. Predicted distance error summary when experimentally accessible NMR parameters included.

Inverse-IMPRESSION Trained on δ^{1}H and δ^{13}C

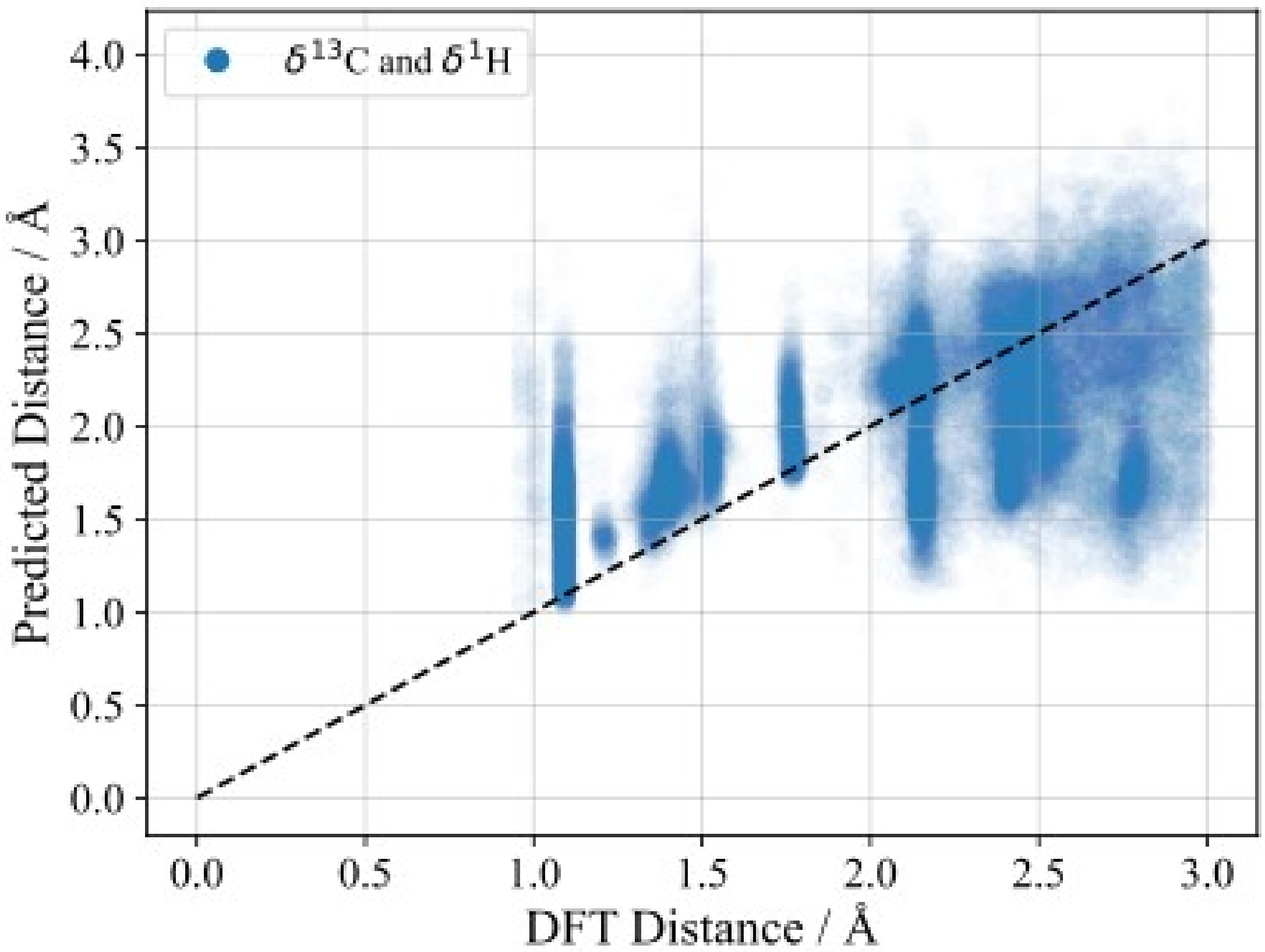

Fig S8. Predicted distances at short range (<3Å) with just δ^{1}H and δ^{13}C chemical shift included.

	MAE / Å	RMSE / Å	Mean Error	Max Error	R^2
All Distances	3.87	5.12	-3.80	27.0	-1.12
Distances<3Hz	0.42	0.53	-0.10	2.52	0.14

Table S4. Predicted distance error summary with just δ^{1}H and δ^{13}C chemical shift included.

Confidence Estimation

Associated confidence scores are generated alongside each inverse-IMPRESSION structure prediction. This confidence metric is calculated according to the agreement between the ground truth input δ^{1}H and δ^{13}C chemical shifts and the forward IMPRESSION-G2-predicted δ^{1}H and δ^{13}C chemical shifts of each candidate. The MAEs of both parameters are normalised and summed as per:

$$\text{Confidence} = 1 - \left[MAE_{\delta 13C} * \frac{10}{220} + MAE_{\delta 1H} * \frac{10}{12}\right] \qquad \textbf{Eq. S6}$$

A series of examples are shown below -- taken from the test set where multiple candidates were generated (either by input feature noising or heteroatom bond enumeration) and the correct structure was successfully identified by confidence scoring. The candidate structure and corresponding confidence score is shown in each case.

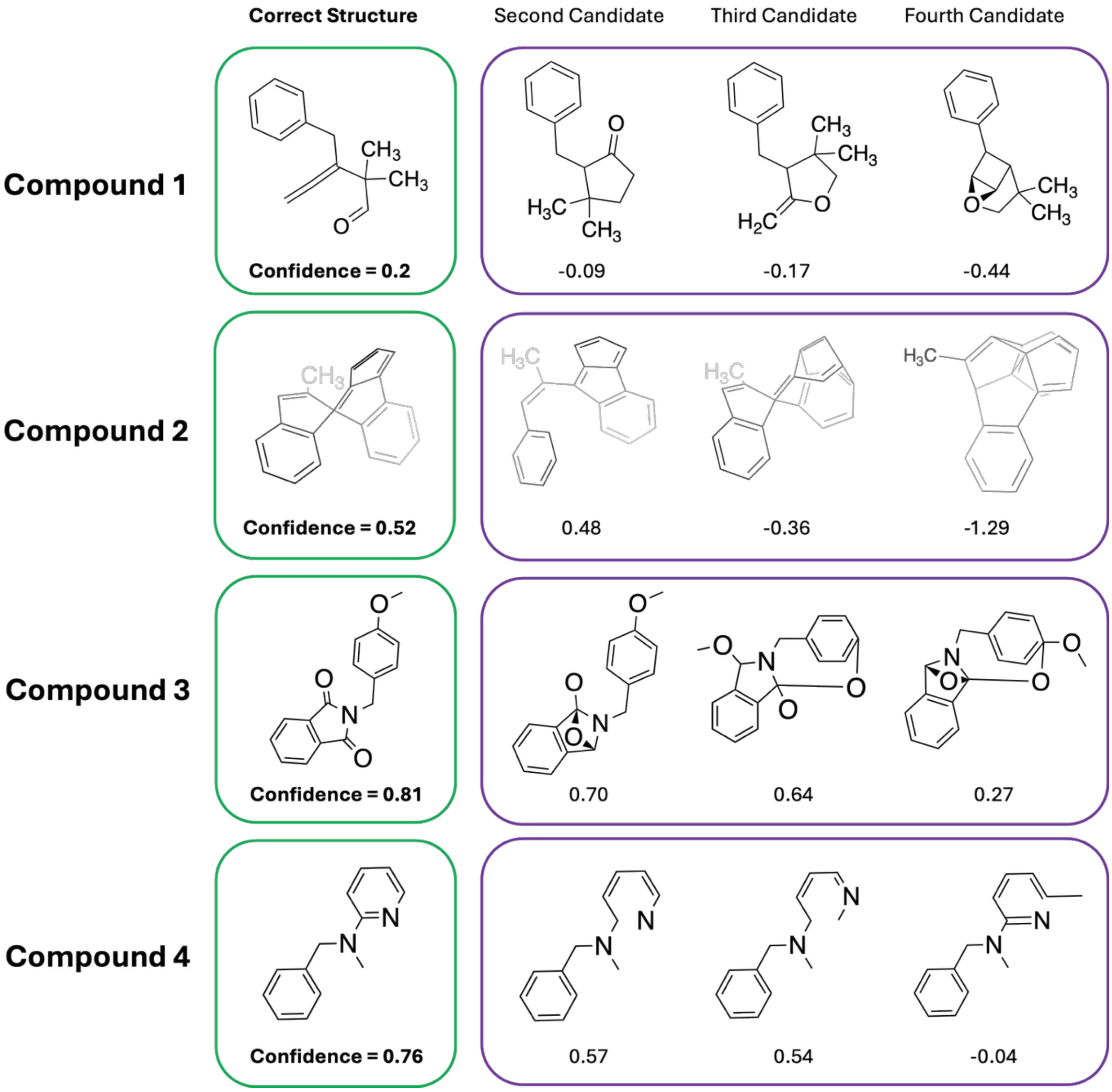

Fig S9. A series of examples: four compounds correctly obtained by inverse-IMPRESSION from experimentally accessible parameters and correctly identified from a series of potential candidates by forward-IMPRESSION confidence scoring.

The >95% success rate observed in identifying correct candidates from a set of multiple possible options demonstrates that this confidence metric is highly proficient in identifying the correct structure *if* it is present. However, since the forward IMPRESSION expected error varies depending on the chemical structure, the distribution of confidence scores for correct and incorrect structures is not perfectly uniform across different compounds. For example, the correct candidate structure of compound 1 has a lower confidence score than incorrect candidates for compounds 2-4. To determine the extent of this feature, a confidence computation was run on every 1-shot prediction of the hold out test set. In general, the

average confidence score gives a strong distinction between correct and incorrect structure predictions. Crucially too, the magnitude of this score is highly sensitive to the degree to which the predicted structure is incorrect (how many incorrect bonds it contains). A statistical analysis of the confidence scores showed that 98.8% of correct answers have a confidence score above 0 and therefore, a predicted structure with a negative confidence can be generally expected to be incorrect. 99.4% of incorrect answers have a confidence score below 0.7, so this can be similarly considered the threshold for a confident correct answer. Otherwise, the confidence threshold can be varied to capture different numbers of candidates, depending on how stringent the test is intended to be. The table provides a guide to help to determine this threshold, based on confidence scores computed for the hold out test set.

Confidence Threshold	Correct Answers Above Threshold	Incorrect Answers Above Threshold
0	98.8%	31.8%
0.1	97.6%	25.9%
0.2	96.4%	20.3%
0.3	94.1%	14.5%
0.4	91.7%	9.3%
0.5	83.3%	5.1%
0.6	70.8%	2.3%
0.7	54.2%	0.6%
0.8	25.6%	0.0%

Table S5. A breakdown of how different confidence score cut-offs capture different proportions of true positives and false positives.

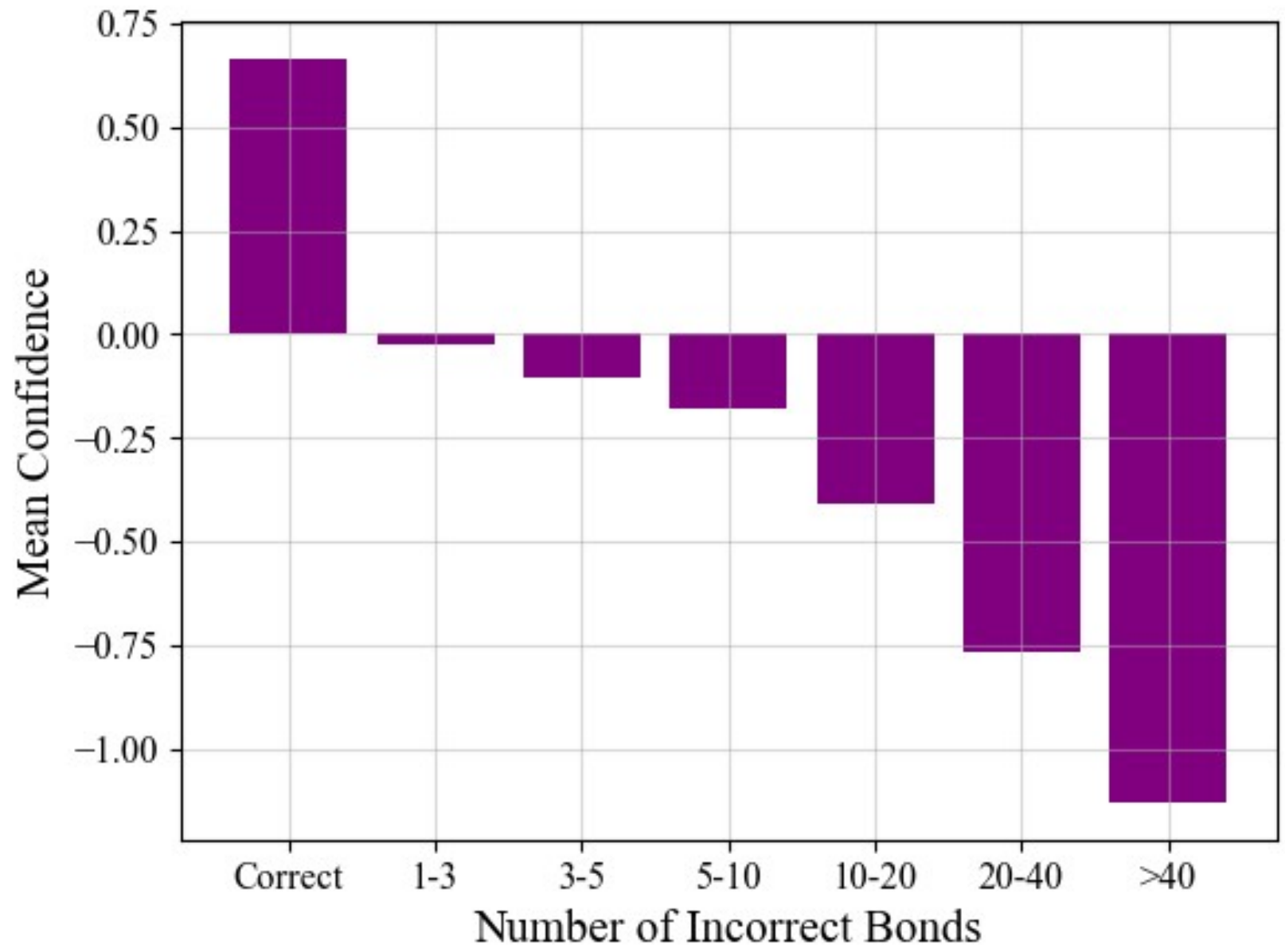

Fig S10. How average confidence score differs between correct structures and structures incorrectly predicted to differing degrees.

Performance Relative to Training Set Size

One solution to improving performance of the models would be to increase the training dataset size. Fig S11 shows model performance improves with increased training set size (at <2.5 Å, given the log normal loss function is intended to bias model training towards such short-range distances). This indicates that performance could be improved further. However, in line with what we have found in previous works, a ten-fold training set increase approximately halves the prediction error (Fig S12). In order to significantly improve the prediction error further, a large investment is required into training set expansion – representing a huge computational burden running DFT and also challenges in terms of training compute resource. Consequently, we are exploring alternative ways to generate and train on substantially larger datasets in this respect.

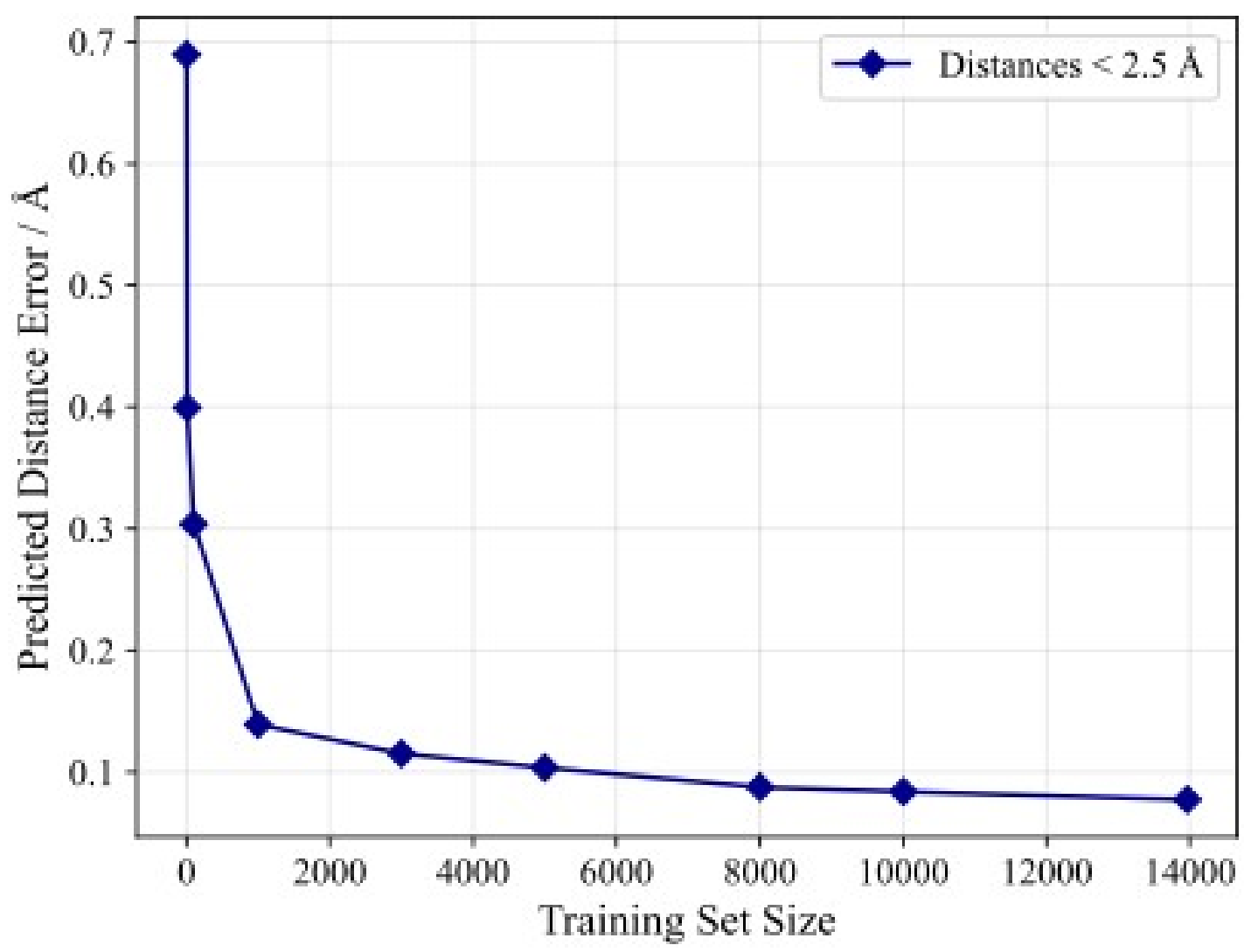

Fig S11. How prediction error at short range changes with training set size.

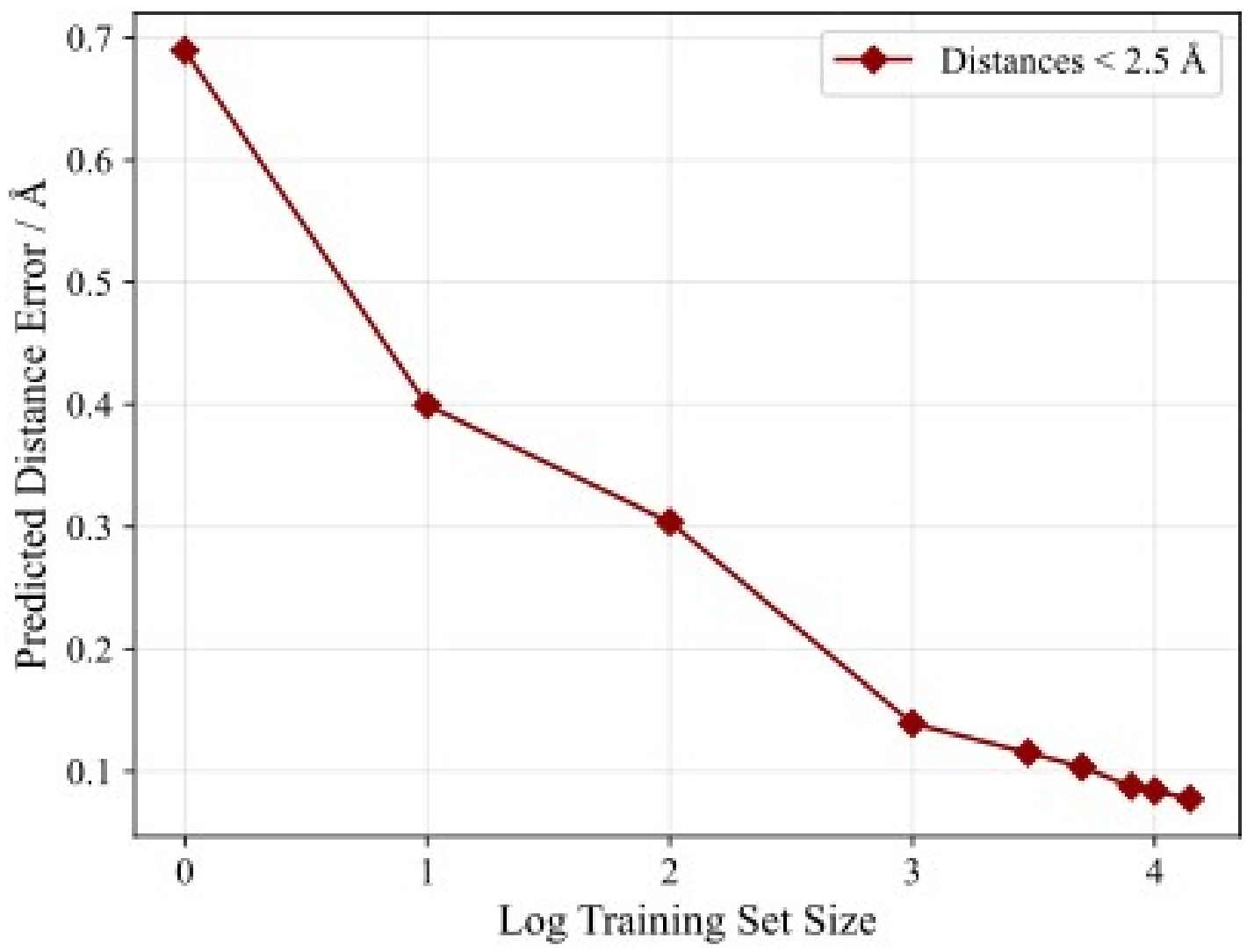

Fig S12. How prediction error at short range changes with training set size on a log scale.

References

1. C. Yiu, B. Honoré, W. Gerrard, J. Napolitano-Farina, D. Russell, I. M. L. Trist, R. Dooley and C. P. Butts, *Chemical Science*, 2025, **16**, 8377-8382.
2. L. A. Bratholm, W. Gerrard, B. Anderson, S. Bai, S. Choi, L. Dang, P. Hanchar, A. Howard, S. Kim and Z. Kolter, *Plos one*, 2021, **16**, e0253612.
3. C. R. Groom, I. J. Bruno, M. P. Lightfoot and S. C. Ward, *Structural Science*, 2016, **72**, 171-179.
4. D. Mendez, A. Gaulton, A. P. Bento, J. Chambers, M. De Veij, E. Félix, M. P. Magariños, J. F. Mosquera, P. Mutowo and M. Nowotka, *Nucleic acids research*, 2019, **47**, D930-D940.
5. OTAVA diversity library – PrimScreen-10 database, https://otavachemicals.com/sdf/les-download/599_366eead837ccf83857cb4fa7e94943ab, (accessed March 2021).
6. C. Steinbeck, S. Krause and S. Kuhn, *Journal of chemical information and computer sciences*, 2003, **43**, 1733-1739.
7. W. Gerrard , L. A. Bratholm , M. J. Packer , A. J. Mulholland , D. R. Glowacki and C. P. Butts , *Chem. Sci.*, 2020, **11** , 508 —515
8. G. Landrum, RDKit: Open-Source Cheminformatics, 2006, https://www.rdkit.org.
9. Y. Guan , S. S. Sowndarya , L. C. Gallegos , P. C. S. John and R. S. Paton , *Chem. Sci.*, 2021, **12** , 12012 —12026
